# Domain-independent deception: a new taxonomy and linguistic analysis

**DOI:** 10.3389/fdata.2025.1581734

**Published:** 2025-09-30

**Authors:** Rakesh M. Verma, Nachum Dershowitz, Victor Zeng, Dainis Boumber, Xuting Liu

**Affiliations:** ^1^Department of Computer Science, University of Houston, Houston, TX, United States; ^2^School of Computer Science and AI, Tel Aviv University, Tel Aviv, Israel; ^3^Department of Computer Sciences, University of Houston, Houston, TX, United States; ^4^InstaBase, San Francisco, CA, United States; ^5^Department of Computer Sciences, University of California, Berkeley, Berkeley, CA, United States

**Keywords:** automatic/computational deception detection, cross-domain, domain-independent, email/message scams, fake news, meta-analysis, opinion spam, phishing

## Abstract

**Introduction:**

Internet-based economies and societies are drowning in deceptive attacks. These attacks take many forms, such as fake news, phishing, and job scams, which we call “domains of deception.” Machine learning and natural language processing researchers have been attempting to ameliorate this precarious situation by designing domain-specific detectors. Only a few recent works have considered domain-independent deception. We collect these disparate threads of research and investigate domain-independent deception.

**Methods:**

First, we provide a new computational definition of deception and break down deception into a new taxonomy. Then, we briefly mention the debate on linguistic cues for deception. We build a new comprehensive real-world dataset for studying deception. We investigate common linguistic features for deception using both classical and deep learning models in a variety of situations including cross-domain experiments.

**Results:**

We find common linguistic cues for deception and give significant evidence for knowledge transfer across different forms of deception.

**Discussion:**

We list several directions for future work based on our results.

## 1 Introduction

History is replete with famous lies and deceptions. Examples include P. T. Barnum, Nicolo Machiavelli, Sun Tzu, Operation Mincemeat, and the Trojan Horse (Levine, 2014). A chronology of deception is included in Levine (2014). More recently, the proliferation of deceptive attacks such as fake news, phishing, and disinformation is rapidly eroding trust in Internet-dependent societies. The situation has deteriorated so much that 45% of the US population believes the 2020 US election was stolen.^1^

Social media platforms have come under severe scrutiny regarding how they police content. Facebook and Google are partnering with independent fact-checking organizations that typically employ manual fact-checkers.

Natural-language processing (NLP) and machine learning (ML) researchers have joined the fight by designing fake news, phishing, and other kinds of domain-specific detectors.

Building single-domain detectors may be sub-optimal. Composing them sequentially requires more time, and composing them in parallel requires more hardware. Moreover, building single-domain detectors means one can only react to new forms of deception after they emerge.

Our goal here is to spur research on *domain-independent* deception. Unfortunately, research in this area is currently hampered by the lack of computational definitions and taxonomy, high-quality datasets, and systematic approaches to domain-independent deception detection. Thus, the results are neither generalizable nor reliable, leading to much confusion.

Accordingly, we make the following contributions:

- We propose a new computational definition and a new comprehensive taxonomy of deception. (We use the unqualified term “deception” for the domain-independent case. When the goals of the deception are unclear, we refer to “lies”).
- We examine the debate on linguistic deception detection, identify works that demonstrate the challenges that must be overcome to develop domain-independent deception detectors, and examine them critically.
- We conduct linguistic analysis of several detection datasets for general cues and find several statistically significant ones.
- We conduct deep learning experiments of deception sets and study correlations in performance for pairs of datasets.

This article is organized as follows: Section 2 presents a new definition of deception. Section 3 introduces our new taxonomy. Section 4 summarizes related work. Sections 5 and 6 describe our experiments, results, and analysis of domain-independent markers for deception. Cross-domain detection results are in Section 7. Finally, Section 8 presents some conclusions and directions for the future. The appendices provide the list of features tested and some preliminary significance testing of cues on four public deception datasets.

## 2 Definition

We first examine a general definition of deception, taken from Galasinski (2000), intended to capture a wide variety of deceptive situations and attacks.

** Definition 1 (Preliminary)**. *Deception* is an intentional act of manipulation to gain compliance. Thus, it has at least one source, one target, and one goal. The source is intentionally manipulating the target into beliefs, or actions, or both, intended to achieve the goals.

Since we are interested in automatic verifiability, we would like to modify this definition of deception and propose one that is computationally feasible. Because intentions are notoriously hard to establish, we will use the effect of exposing the manipulation/goals instead.

Our revised definition is the following:

Definition 2 (Deception). *Deception* is an act of manipulation designed to gain compliance such that, exposing the manipulation or the goal(s) of compliance significantly decreases the chance of compliance. Thus, it has at least one source, one target, and one goal. The source is manipulating the target into beliefs, or action, or both, intended to achieve the goals.

One might argue that the goals of deception should be harmful to an individual or organization. However, this would necessitate either a computational definition of harm or a comprehensive list of potential harms, which could be checked computationally and is, therefore, a less desirable alternative.

To formalize our definition, we borrow from the language of Markov decision processes. Let *A* be an action taken by an actor, and let *C* be a desired compliance state. We use *K*(*A, T*) to denote the action *A* plus the full and truthful explanation of the actor's *relevant* private information to target *T*. We formalize (computational) deception using conditional probabilities as follows:

Definition 3 (Computational Deception—Formalized). An action *A* *deceives* target *T* if


P(C |K(A,T))<P(C |A).

Moreover, we can quantify the degree to which *A* is deceptive by the amount θ, where 0 ≤ θ ≤ 1.

Definition 4 (Computational Deception—Quantified). An action *A* θ*-deceives* target *T* if


P(C |K(A,T))≤P(C |A)-θ.

In practice, practitioners can apply this by exposing the manipulation and/or goals and measuring the change in compliance rates. For example, a Florida woman recently sued Kraft alleging that the “ready in $3\frac{1}{2}$ min” on the label of their microwavable Velveeta Shells & Cheese is deceptive. To determine whether the claim is, in fact, deceptive, a researcher could present the product by itself to one group of random consumers and the product with an explanation that the $3\frac{1}{2}$ min does not include the time to add water to another group. If there is a statistically significant decrease in purchases (which is the desired compliance) for the group with the explanation, then the claim is deceptive.

There is some work on finding out how good humans are at detecting certain kinds of deceptive attacks. For the detection capabilities of automatic detectors on specific domains of deception, one can look at surveys on fake news detection (Sharma et al., 2019; Zhou et al., 2019, 2020) and phishing detection (Das et al., 2020).

## 3 Taxonomy and examples

In this section, we give a new taxonomy for deception and some examples to illustrate it. Note that this taxonomy is intended to be comprehensive and capture all nuances of deception, which means also the legal aspects when the source of the deception is being charged with a crime for example. Hence, this taxonomy will take into account the intent of the source in contrast with the above section.

There have been a few attempts at constructing taxonomies for fake news, phishing, or other forms of deception. Molina et al. (2021) give a taxonomy of *fake news* with four dimensions: message and linguistic, sources and intentions, structural, and network. Kapantai et al. (2021) conducted a systematic search for papers proposing taxonomies for disinformation and synthesized a taxonomy with three dimensions: factuality, motivation, and verifiability.

No one, to our knowledge, has given a comprehensive taxonomy of real-world deception.

### 3.1 The new taxonomy

**Figure 1:**
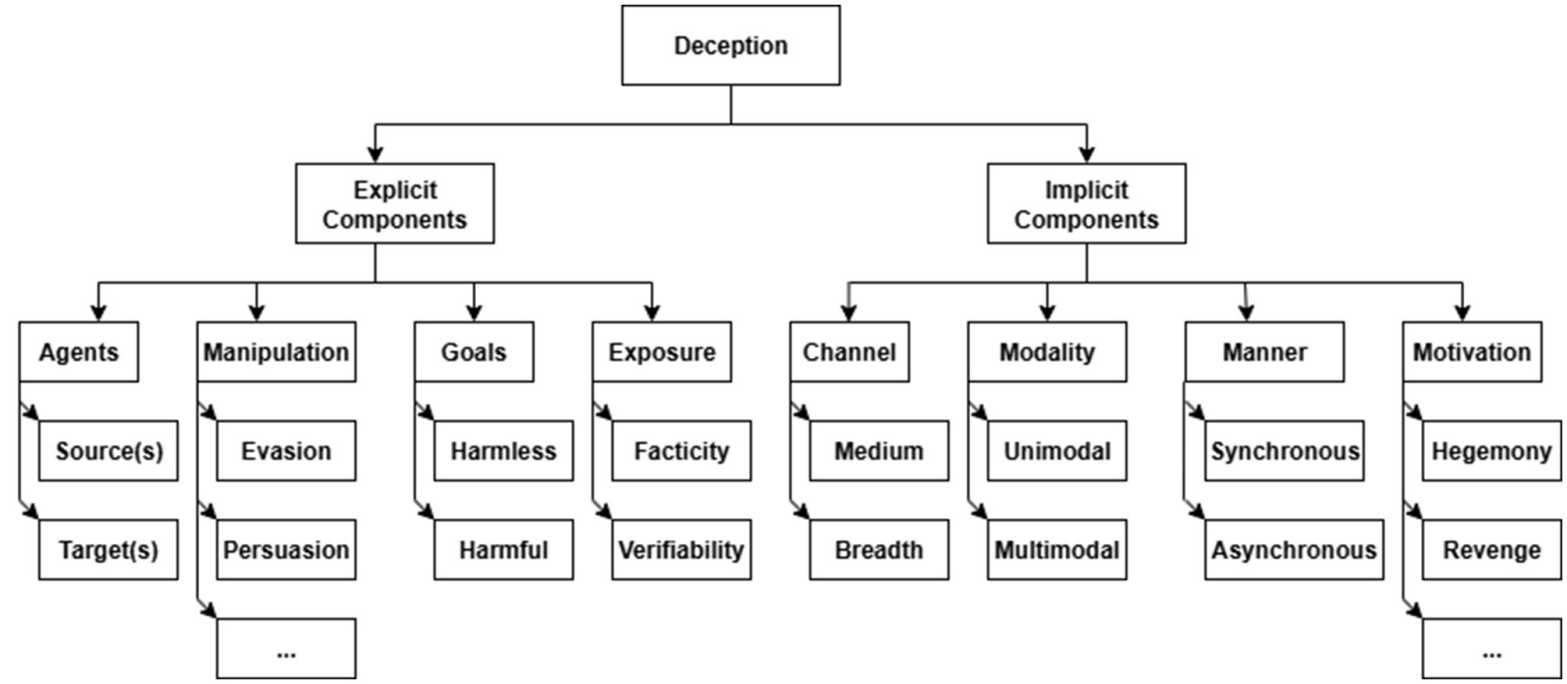
Proposed deception taxonomy—the full manipulation (or stratagem) and motivation subtrees are not shown.

We put forward a multi-dimensional taxonomy (Figure 1). Under our definition, deception explicitly involves four elements: (1) agents: the sources, and the targets; (2) stratagems for manipulation; (3) goals; and (4) threat/mechanisms of exposure. These explicit elements can be further broken down as follows:

1) *Agents*. Rowe (2006) calls this category “participant,” and he further elaborates this into: (a) agent, who initiates the action, (b) beneficiary, who benefits, (c) object, what the action is done to, and (d) recipient, who receives the action. Rowe also includes experiencer (“who senses the action”) and instrument (“what helps accomplish the action”) components in this category, but we include them in the Channel category below.
1a) Sources. This includes human (individual or group), bot, etc., or mixed, in other words, combinations such as a human assisted by a bot. This Sources category includes initiators and beneficiaries.
1b) Targets. This includes humans (individual or group), automatic detectors, or both. For example, spam targets automatic detectors, and phishing targets humans, but needs to fool automatic detectors also. This Targets category includes the objects and the recipients.
2) *Stratagems*. The stratagem subtree in the taxonomy includes two sub-taxonomies for persuasion and action, which we discuss below. We believe that persuasion is fundamental to deception since its goal is to change the reasoning of the target(s), with the deception's end goal of compliance. The action taxonomy is adapted from Rowe (2006). It includes space, time, causality, quality, essence, and speech-act theory, which specifies the external and internal preconditions for the action. The persuasion taxonomy combines Cialdini (2006) and Da San Martino et al. (2023).
3) *Goals*.
3a) Harmless: satire, parody, satisfying participation, as in a laboratory experiment where participants may be asked to lie, etc.
3b) Harmful. This includes a wide range of objectives, such as stealing money or identity information, malware installation, manipulation of votes, planting fear, sowing confusion, initiating chaos, gaining an unfair edge in a competition (e.g., swaying opinions and preferences on products), persuading people to take harmful actions, winning competitions/games, etc. We avoid the terms defensive and offensive since they are dependent on the perspective of the participants/agents.
4) *Exposure*.
4a) Facticity. Can we establish whether it is factual or not? For example, currently, we are unable to establish the truth or falsity of utterances such as, “There are multiple universes in existence right now.”
4b) Verifiability. Assuming facticity, how easy or difficult it is to verify whether it is legitimate or deceptive? Here, we are interested in machine or automatic verification. If a simple machine-learning algorithm can detect it with high recall and precision, we will deem it easy.

In addition, there are four implicit concepts in the taxonomy: (1) motivations behind the goals; (2) communication channels or media; (3) modality of deception; and (4) manner or timeliness of the exchange.

1) *Motivation*. This is the rationale for the goals. The agents involved and their characteristics reveal the underlying motivations, which could be political hegemony (nation-states), religious domination, revenge (disgruntled employee), ideological gains, money, control, power, etc.
2) *Channel*. This dimension includes two aspects:
2a) Breadth: Whether the targets are a few specific individuals or detector types or broad classes of people/categories of detectors.
2b) Media. How the deceptive capsule is conveyed to the target. Media also includes the experiencer and instrument components of Rowe (2006).
3) *Modality*. This dimension refers to the presentation of deceptive content. It includes:
3a) Unimodal. This includes only one type of modality such as (a) gestural (i.e., body language is used to deceive), (b) audio (a.k.a. verbal), (c) textual (e.g., SMS/email), and (d) visual (e.g., images or videos).
3b) Multimodal: combinations of different modalities. For example, audio-visual has both speech and visual components but lacks face-to-face communication in which gestures could facilitate deception.
4) *Manner/Timeliness*.
4a) Interactive/synchronous. A real-time interview or debate is an interactive scenario.
4b) Non-interactive/asynchronous. An Amazon Mechanical Turker typing a deceptive opinion or essay is a non-interactive one. An asynchronous interaction can have multiple stages or steps some (but not all) of which may be synchronous.

#### 3.1.1 Stratagems

Rowe's (2006) approach is based on linguistics. He states, “Each action has associated concepts that help particularize it, and these are conveyed in language by modifiers, prepositional phrases, participial phrases, relative clauses, infinitives, and other constructs.” These associated concepts are called “semantic cases” Fillmore, (1968) in analogy to the syntactic cases that occur in some languages for nouns. Rowe claims that “every deception action can be categorized by an associated semantic case or set of cases.” However, there is no canonical list of semantic cases in linguistics. Rowe prefers the detailed list from Copeck et al. (1992), which he supplements with two important relationships from artificial intelligence, the upward type-supertype and upward part-whole links, and two speech-act conditions from Austin (1975), to get 32 cases altogether. However, since we include his “participant” category in the Agents and Channel categories, we have only 26 subcategories in the Stratagems category.

1. Space, which consists of: (a) direction, of the action, (b) location-at, where something occurred, (c) location-from, where something started, (d) location-to, where something finished, (e) location-through, where some action passed through, and (f) orientation, in some space.
2. Time, which is subdivided into: (a) frequency of occurrence of repeated action, (b) time-at, time at which something occurred, (c) time-from, the time at which something started, (d) time-to, the time at which something ended, and (e) time-through, the time through which something occurred.
3. Causality, which consists of: (a) cause, (b) contradiction, what this action opposes if anything, (c) effect, and (d) purpose.
4. Quality, which is sub-divided into: (a) accompaniment, an additional object associated with the action, (b) content, what is contained by the action object, (c) manner, the way in which the action is done, (d) material, the atomic units out of which the action is composed, (e) measure, the measurement associated with the action, (f) order, with respect to other actions, and (g) value, the data transmitted by the action (the software sense of the term).
5. Essence, which consists of: (a) supertype, a generalization of the action type, and (b) whole, of which the action is a part.
6. Speech-act theory, which is sub-divided into: (a) an external precondition on the action and (b) an internal precondition on the ability of the agent to perform the action.

#### 3.1.2 Persuasion

We summarize the persuasion taxonomy in Table 1. For this taxonomy, we adapt the SemEval 2023 Persuasion Task's categories (Da San Martino et al., 2023), and Cialdini's (Cialdini, 2006) persuasion principles, which are essentially persuasion techniques or strategies. The persuasion strategies taxonomy of Guerini et al. (2007) is orthogonal to this taxonomy since their definition of persuasion is broader than ours, but we do include their specific strategies under techniques.

**Table 1:** Persuasion taxonomy, adapted from Da San Martino et al. (2023), is a sub-taxonomy in the deception taxonomy.

**Category**	**Description**
Justification	An argument made of two parts: a statement and a justification
Simplification	A statement is made that excessively simplifies a problem, usually regarding the cause, the consequence or the existence of choices
Distraction	A statement is made that changes the focus away from the main topic or argument
Call	The text is not an argument but an encouragement to act or think in a particular way
Manipulative wording/images	Specific language/imagery is used or a statement is made that is not an argument, and which contains words/phrases that are either non-neutral, confusing, exaggerating, etc., to impact the reader, for instance emotionally
Attack on reputation	An argument whose object is not the topic of the conversation, but the personality of a participant, his experience and deeds, typically to question and/or undermine his credibility

The techniques used for each category are as follows (30 in total):

- Justification: appeal to popularity, appeal to authority/expert, appeal to values [or commitment (Cialdini, 2006)], appeal to fear/prejudice, reciprocity (Cialdini, 2006) [or goal balance (Guerini et al., 2007)], scarcity (Cialdini, 2006), reward, appeal to relevant empirical evidence, relevant statistics, and relevant examples.
- Simplification: causal oversimplification, false dilemma or no choice, and consequential oversimplification.
- Distraction: straw man, red herring (includes irrelevant empirical evidence, statistics or examples), whataboutism, flag waving, and liking (Cialdini, 2006).
- Call: slogans, social proof (Cialdini, 2006), appeal to time, and conversation killer.
- Manipulative wording/images: loaded language/images, repetition, exaggeration or minimization, and obfuscation—vagueness or confusion.
- Attack on reputation: name calling or labeling, doubt, guilt by association, appeal to hypocrisy, questioning the reputation.

To the best of our knowledge, we are the first to give detailed taxonomies for persuasion and strategem in this context and we are the first to use the following dimensions in a taxonomy of deception: target, persuasion, goal, dissemination, and timeliness. We add these to give a comprehensive view of deception, to aid in domain-independent deception detection, and to clarify and classify deception in all its different manifestations. Such a comprehensive taxonomy will provide a solid foundation on which to build automatic and semi-automatic detection methods and training programs for the targets of deception.

### 3.2 Examples

To demonstrate the applicability of this taxonomy, we give three examples. More discussion of stratagems and examples of cyber deception can be found in Rowe (2006).

Phishing is when attackers pretend to be from reputable companies to trick victims into revealing personal information. The agents are the attackers as initiators and the targets are the Internet/email users. The harmful goals include information or malware installation. Establishing facticity is difficult if the attacker is determined. The medium is the Internet/email. The breadth is high for phishing and narrower for spear phishing. The modality is text for phishing and audio for vishing. Images may also be used in phishing emails. The manner is non-interactive for phishing and interactive for vishing. Deliberate falsification and persuasion techniques such as authority, social proof, and reward or loss claims are employed in the stratagem.

Fake news is manufactured and misleading information presented as news. Here, the harmful goals include swaying opinion, sowing unrest, and division. The sources could be individuals, organizations, or nation-states. The breadth could vary depending on how deep-pocketed and determined the source(s) is (are). The modality could be text, audio, images, or video. The manner is asynchronous. Fake news could employ a range of techniques in the action component of the stratagem: from deliberate falsification to evasion and the persuasion component could include authority, social proof, etc.

Fake reviews are reviews designed to give consumers a false impression of a product or business. The harmful goal is to convince consumers to buy their product or avoid a competitor. The sources could be humans, bots, or their combinations. The targets are potential customers as well as the platform's fake review detector. The breadth is thus a broad range of people. While most fake reviews use only texts, deliberate attacks could be multi-modal, adding visuals and/or audio. Falsification and social proof are the main stratagems. Facticity and verifiability could vary depending on the stratagems used. The manner is asynchronous.

## 4 Related work

Deception has a vast social science literature. Hence, we focus on the most closely related work on computational deception, which can be categorized into taxonomies, datasets, detection, and literature reviews. Of the latter, we focus here on reviews of linguistic deception detection. The DBLP^2^ query “domain decepti”^3^ gave 43 matches of which 21 were deemed relevant.

** Remark 1**. Unfortunately, previous researchers have generally left the term “domain” undefined. In Glenski et al. (2020), different social networks, such as Twitter and Reddit, are referred to as domains. Hence, terms such as “cross-domain deception” in previous work could mean that the topics of essays or reviews are varied whereas the goals could stay pretty much the same.

### 4.1 Taxonomies

Whaley and Aykroyd (2007) gave a taxonomy of perception in which deception was defined succinctly as “other-induced misperception.” The full definition given in Whaley and Aykroyd (2007) is: “Any attempt—by words or actions—intended to distort another person's or group's perception of reality.” In Bell and Whaley (2017), two groups were introduced as essential for deception: simulation (overt, showing the false) and dissimulation (covert, hiding what is real). They introduced three simulation techniques: mimicking, inventing, and decoying, and three dissimulation techniques: masking, repackaging, and dazzling.

Dunnigan and Nofi (2001) gave a taxonomy of deception in the military context. This included concealment, camouflage, disinformation, lies, displays, ruses, demonstrations, feints, and insight.

The most comprehensive previous taxonomy of deception, to our knowledge, is proposed in Rowe (2006). It is inspired by linguistic case theory and includes 32 cases which are grouped into seven categories: space (six cases), time (five cases), participant (six cases), causality (four cases), quality (seven cases), essence (two cases), speech-act theory (two cases). Analyzing this taxonomy, we find that, except for the participant category, all the other categories fit neatly into the stratagems class for deception in our taxonomy.

More recently, a few researchers have proposed more specialized taxonomies for what they call defensive deception (Oluoha et al., 2021; Pawlick et al., 2019; Pawlick and Zhu, 2021). Some folksy and psychological taxonomies are given in Druckman and Bjork (1992).

### 4.2 Datasets

Several datasets have been collected for studying lies. However, researchers have not carefully delineated the scope by considering the goals of the deception. There is also another potentially more serious issue: Some datasets are constructed by asking participants to lie in a laboratory setting, where there are no consequences and no incentive to lie. We will refer to them as *Lab Datasets*. Others are constructed by collecting samples of real attacks. We call them *Real-World Datasets*. Finally, there are some datasets in which data from laboratory settings are combined with real-world attack samples. We call them *Mixed Datasets*.

Lab Datasets include Zhou et al. (2004), wherein students were paired and one student in each pair was asked to deceive the other using messages. In Perez-Rosas (2014), researchers collected demographic data and 14 short essays (seven truthful and seven false) on open-ended topics from 512 Amazon Mechanical Turkers (AMT). They tried to predict demographic information and facticity. We refer to this as the *Open-Lies* dataset. In Pérez-Rosas and Mihalcea (2014), researchers collected short essays on three topics: abortion, best friend, and the death penalty by people from four different cultural backgrounds. In Capuozzo et al. (2020), truthful and deceptive opinions on five topics are collected in two languages (English and Italian). See Ludwig et al. (2016) for more such efforts.

Next, we consider real-world datasets, where the goals may be information, disruption, financial, or psychological. Here, we have several datasets for fake news detection (Raponi et al., 2022),^4^ opinion spam (a.k.a. fake reviews) detection (Ren and Ji, 2019), phishing (Verma et al., 2019), and a company's reward program (Ludwig et al., 2016).

Some researchers have mixed data obtained from laboratory settings with non-laboratory data, such as reviews obtained from forums. For example, in Hernández-Castañeda et al. (2017), researchers analyzed three datasets: a two-class, balanced-ratio dataset of 236 Amazon reviews, a hotel opinion spam dataset consisting of 400 fabricated opinions from AMT plus 400 reviews from TripAdvisor (likely to be truthful), and 200 essays from Pérez-Rosas and Mihalcea (2014). In Xarhoulacos et al. (2021), researchers studied a masking technique on two datasets: a hotel, restaurant, and doctor opinion spam dataset and the dataset from Pérez-Rosas and Mihalcea (2014). In Cagnina and Rosso (2017), in-domain experiments were done with a positive and negative hotel opinion spam dataset, and cross-domain experiments were conducted with the hotel, restaurant, and doctor opinion spam dataset.

A few works have developed domain-independent deception datasets in our sense, wherein the goals of deception can be quite different. In Rill-García et al. (2018), researchers used two datasets: the American English subset consisting of a balanced-ratio 600 essays and transcriptions of 121 trial videos (60 truthful and 61 deceptive), which we call Real-Life_Trial. In Vogler and Pearl (2020), three datasets were used: positive and negative hotel reviews, essays on emotionally-charged topics, and personal interview questions. In Xarhoulacos et al. (2021), multiple fake news datasets, a COVID-19 dataset, and some micro-blogging datasets were collected and analyzed. In Shahriar et al. (2021), researchers collected fake news, Twitter rumors, and spam datasets. (Spam is essentially advertising. Deception is employed to fool automatic detectors rather than the human recipient of the spam. We focus on human targets.) They applied their models trained on these datasets to a new COVID-19 dataset. In Yeh and Ku (2021), seven datasets were collected (Diplomacy, Mafiascum, Open-Domain, LIAR, Box of Lies, MU3D, and Real-Life_Trial) and analyzed using LIWC categories, without claiming domain independence or cross-domain analysis. However, their datasets do involve different goals. LIAR, for instance, includes political lies with the goal of winning elections, whereas the lies in Real-Life_Trial have other goals, and Diplomacy/Mafiascum are about winning online games. In Feng et al. (2012), four datasets were collected: trip-advisor gold, a balanced hotel reviews dataset of 800 reviews introduced in Ott et al. (2011), trip-advisor heuristic, another balanced reviews dataset of 800 reviews collected by the authors, a third 800 review Yelp dataset of uncertain ground-truth collected by the authors, and the 296 essays on three topics dataset of Mihalcea and Strapparava (2009). They show that features based on CFG parse trees along with unigrams performed the best on these datasets.

Thus, we still lack large, comprehensive datasets for deception that have a wide variety of deceptive goals.

### 4.3 Detection

Deception detection in general is a useful and challenging open problem. There have been many attempts at specific applications such as phishing and fake news. On phishing alone (query: phish), there are 2,200+ DBLP results, including over 70 surveys and reviews. Similarly, there are 1,100+ papers on scams (query: scam, not all of them are relevant, since many occurrences are part of acronyms such as SCAMP), 100+ on opinion spam, 200+ on fake reviews, and 2,600+ on fake news.^5^

A soft domain transfer method is proposed in Shahriar et al. (2022). They found that partial training on tweets helped in phishing and fake news detection. In Panda (2022) and Panda and Levitan (2023), the authors study deception detection across languages and modalities. Other works on domain-independent deception detection have been discussed above under datasets.

### 4.4 Reviews on linguistic markers

Recently, Gröndahl and Asokan (2019) conducted a survey of the literature on deception. They defined implicit and explicit deception, focused on automatic deception detection using input texts, and then proceeded to review 17 papers on *linguistic* deception detection techniques (explicit deception is when the deceiver explicitly mentions the false proposition in the deceptive communication). These papers covered two forms of deception: (a) dyadic pairs in the laboratory, where one person sends a short essay or message to another (some truthful and some lies), and (b) fake reviews (a.k.a. opinion spam). Based on their analysis of the literature on laboratory deception experiments and the literature on opinion spam, they concluded that *there is no linguistic or stylistic trace that works for deception in general*. Similarly, the authors of Vogler and Pearl (2020) assert that extensive psychology research shows that “a generalized linguistic cue to deception is unlikely to exist.” We collectively refer to Gröndahl and Asokan (2019), Fitzpatrick et al. (2015), and Vogler and Pearl (2020); Vrij (2008) as the *Critiques*.

As opposed to the critiques, the meta-analyses by DePaulo et al. (2003) and Hauch (2016) did find small markers of deception in the studies they examined despite analyzing studies of specific forms or situations of deception, not general domain-independent datasets. Similarly, the following papers all point to evidence for cross-domain deception detection: Rill-García et al. (2018), Shahriar et al. (2021), Vogler and Pearl (2020), Xarhoulacos et al. (2021), and Yeh and Ku (2021). These researchers created so-called “domain-independent datasets,” which consist of two or three kinds of attacks and developed features and techniques for deception detection across the collected domains.

We believe that a deeper investigation/analysis of the linguistic cues for deception debate is needed, for the simple reason that none of the above works created a comprehensive dataset of different forms of deceptive attacks and analyzed it.

## 5 Linguistic cues/analysis

Because of the problems enumerated above, we collect and analyze datasets for domain-independent linguistic cues to tackle: (1) the ground truth problem for deception detection and (2) evidence of linguistic cues for deception across domains.

A *ground truth* is something that is known to be correct, but this information is difficult to obtain, so we need models that do not rely on having too much ground truth data. Our approach is to focus on using linguistic information from the text. For the second challenge, we try to find universal linguistic markers for deception by looking for features that behave similarly across domains. We hope that an ML model built with these features could generalize across domains (Gokhman et al., 2012).

### 5.1 Datasets

We summarize our deception domains and scenarios below. We focus on real-world datasets.

In the *product review* domain, we use the Amazon reviews dataset mentioned above (Garcia, 2019).

In the *job scam* domain, we identify fraudulent job listings. Our dataset contains the bodies of 13,735 legitimate and 608 fraudulent job listings.

In the *phishing* domain, we distinguish between legitimate emails and phishing emails. Our dataset contains the bodies of 9,202 legitimate and 6,134 phishing samples. The IWSPA-AP dataset analyzed above is a subset of this dataset.

In the *political statement* domain, we determine the truthfulness of claims made by US political speakers. Our dataset contains 7,167 truthful and 5,669 deceptive statements evaluated by PolitiFact.

In the *fake news* scenario, we distinguish between legitimate and fake news. Here, we use the WELFake dataset (Verma et al., 2021).

We analyzed each dataset for any artifacts of data collection and cleaned them to remove such artifacts. The cleaning procedures include two parts: text removal and text cleaning. We then sanitize the texts using the methods discussed in Zeng et al. (2022). We remove meta-data in emails and source leaks in news and replace HTML break tags with new lines. In addition, the authors of Zeng et al. (2022) found that the provided labels in WELFake Verma et al., (2021) are flipped, so we flip its labels as a final cleaning step. We are making the combined, cleaned dataset available on Zenodo.^6^

### 5.2 Sources for linguistic cues

Function words (FW) are words that express a grammatical relationship between words in a sentence. Unlike content words, function words such as “when,” “at,” and “the” are independent of specific domains. Function words and *n*-grams are useful for many text classification tasks, including author gender classification, authorship attribution (Argamon and Levitan, 2005), and deception detection (Siagian and Aritsugi, 2020). To gain an insight into the transfer of knowledge between domains, we utilized three types of explainable features: function words, part-of-speech (POS) tags of function words, and engineered linguistic features. POS tags were used to determine whether a word was a function or a content word; the content words were then removed. The last experiment utilized 151 engineered linguistic features (13+55+86 − 3 duplicates removed by the colinearity check below).

The engineered features are drawn from three sources. Linguistic Inquiry and Word Count (Boyd et al., 2022), a popular source of features in the NLP literature, was the source of 86 features. The authorship attribution paper (Fabien et al., 2020) was the source of 55 features. Thirteen features were collected from two papers, one on deception (Zhou et al., 2004) and the other on fake news (Verma et al., 2021), after significance testing using *t*-tests with and without the Bonferroni-Holm correction of *p*-values.

The initial significance testing of 27 linguistic features from the two papers (Zhou et al., 2004; Verma et al., 2021) on four public datasets is described in Appendix A. Appendix B describes an analysis of function word *n*-grams on the same datasets as in Appendix A. A complete source-wise list of the 55 features from Fabien et al. (2020) and 86 features from Boyd et al. (2022) is in Appendix C. Function words as features for deception have been studied before, in Siagian and Aritsugi (2020), for example. We also experimented with the part-of-speech tags of function words.

### 5.3 Results of feature analysis

We used the Stanza (Qi et al., 2020) POS tagger and OntoNotes Release 5.0/Penn Treebank (Marcus et al., 1993) tagset in all experiments involving POS tags. This tagset builds on top of the original Penn Treebank and adds seven new tags:

ADD—Email, AFX—Affix, HYPH—Hyphen, NFP—Superfluous punctuation, UH—Interjection, SP—Space, and XX—Unknown.

Due to the parser's limitations, several samples of text that had a length more than one million characters had to be discarded. We did not remove stop words or further alter the data in any manner. Function words and their respective POS tags were separately vectorized as word unigrams using the tf-idf scheme. The raw texts were processed and vectorized identically and used as a baseline. The motivation behind it was to (i) understand whether it is possible to achieve similar results while using only a few non-domain-specific features that are highly indicative of deception and (ii) investigate the impact of content words on deception through the contrast between the baseline and function words.

For each dataset, and for each set of features, we applied three techniques to select the most relevant features. First, a random forest algorithm (Breiman, 2001) was used, which allowed us to rank features by their importance. The least important ones were removed under the condition that the out-of-bag accuracy on the validation set either increased or remained the same after removing the features. Next, we applied scipy's (Virtanen et al., 2020) single linkage hierarchical clustering (Gower and Ross, 1969) with Spearman's correlation (Spearman, 1904) as the measure of feature colinearity. Features exhibiting a high degree of colinearity were removed with their redundancy validated in the same manner as with the first technique. Finally, taking the remaining features, we applied Hyperopt's (Bergstra et al., 2013) feature selection and the eXtreme Gradient Boosting algorithm (Chen and Guestrin, 2016) with SHAP (Lundberg and Lee, 2017) as a metric of each feature's contribution to the overall model performance. Ultimately, the aforementioned approach produced a subset of the features for each of the five datasets. A total of 81 linguistic, 28 function word POS, and 61 function word features were selected; 50/81, 22/28, and 29/61 were shared with at least one other dataset.

For our analysis of the potential for knowledge transfer, any feature unique to a dataset was removed, leaving only those significant for at least two datasets and therefore being of interest for understanding of transfer between domains. The relationships of function words, function words' POS tags, and engineered linguistic features across datasets are depicted in Table 2. Several trends can be noticed from this table. For example, all five datasets share 6+10+1 = 17 common features, and the fake news, job scams, and phishing datasets have a total of 31 features in common. In addition, the subset {F, J} has 35 common features, and {J, P, Pr, Ps} has 26 common features. Job scams and phishing together have 43 common features. Similarly, we see that deceptive attacks can be differentiated using features such as “to,” personal pronouns, singular present verb forms, modals, and adverbs (compare with the quote from Rowe, 2006 in Section 3.1.1). The richness, possessive ending, and interjection features are significant for fake news, job scams, and phishing. Fake news and product reviews have many significant LIWC features.

**Table 2:** Unified feature table showing common features across subsets of domains.

**Subset**	**Function words**	***N***	**FW POS tags**	***N***	**Eng'd linguistic features**	***N***
All	And, In, Is, Of, On, The	6	CC, CD, DT, IN, MD, PRP, RB, TO, VBP, VBZ	10	per_cap	1
F, J, P, Pr	This, You	2		0		0
F, J, P, Ps		0	RP, VB, WDT, WP, WRB	5		0
F, J, Pr, Ps	Are	1		0		0
F, P, Pr, Ps		0	VBD	1		0
J, P, Pr, Ps	for, to	2		0	Dic, f_b, f_g, per_digit, richness	5
F, J, P	at	1	POS, UH	2	cert, f_e_2, function, sen_len	4
F, J, Pr		0		0	Period	1
F, P, Pr		0		0	Paus	1
F, P, Ps		0	EX, VBN	2		0
F, Pr, Ps	It, That, Would	3		0		0
J, P, Ps	From, Our	2		0		0
J, Pr, Ps	As, With	2		0		0
P, Pr, Ps	not	1		0	conj, f_f, modi	3
F, J		0	VBG	1	Apostro, Comm	2
F, P	all, had	2		0	f_e_0, f_e_1, f_e_3, f_e_7, Sens	5
F, Pr		0		0	Adverb, allPunc, Analytic, f_e_8, focuspast, ipron, len_text, OtherP, Pronoun, sen_len	10
F, Ps	He	1		0		0
J, P		0	ADD	1	f_c, f_o, f_v, f_w, Socrefs	5
J, Pr	Be, or	2		0	Allure, Article, Lifestyle	3
J, Ps	we	1		0		0
P, Pr	Me	1		0	avg_len, f_d, f_i, f_s, f_t, f_y, Selfref	7
P, Ps		0		0	f_1, f_p	2
Pr, Ps	They, Was	2		0	Quantity	1

FW, function words; F, fake news; J, job scams; P, phishing; Pr, product reviews; Ps, political statements; Feature sets are inherited downward from supersets to subsets. **N** is the number of features.

#### 5.3.1 Linguistic overlap across deceptive domains

The observed overlap in common features across domains suggests a shared linguistic substrate of deceptive or persuasive communication. Many of the common function words (e.g., *you, this, are*, and *that*) are tied to reader-directed or modal constructions, which have been found to correlate with manipulative intent (Zhou et al., 2004; DePaulo et al., 2003).

Part-of-speech tags such as PRP, VB, TO, and IN reflect structural scaffolding typical in persuasive or fraudulent writing. These tags often co-occur in imperative or passive constructions that are used to command attention or obscure agency (Ott et al., 2011). For instance, phishing emails and fake job offers rely on templates such as “to confirm your account...” or “you are selected...”, which map to these tags.

Engineered features such as avg_len, sen_len, and focuspast point to reduced syntactic complexity and temporal distancing, both recognized as cues in deceptive text (Hancock et al., 2007). Shorter messages and generic phrasing enable broad applicability and reduce the chance of contradiction.

Clusters such as {F, J, P} tend to involve transactional deception (e.g., scams), while overlaps in {Pr, Ps, P} suggest persuasive manipulation.

Datasets that share a significant number of features are good candidates for domain adaptation; however, the performance of a model using a potentially limited set of features shared across tasks must remain robust. To this end, we combined previously selected linguistic, function words, and function word POS features that were shared by two or more datasets. This resulted in a final set of 91 features. Upon further applying feature selection, the number of significant features of all three types shared among datasets has been reduced to 45.

To evaluate the features' performance, we used a random forest classifier with five-fold cross-validation. The model hyperparameters were set to 50 trees with the leaf nodes of five samples, and 50% of the features were considered on each split. Gini impurity was used as a criterion of split quality.

**Figure 2:**
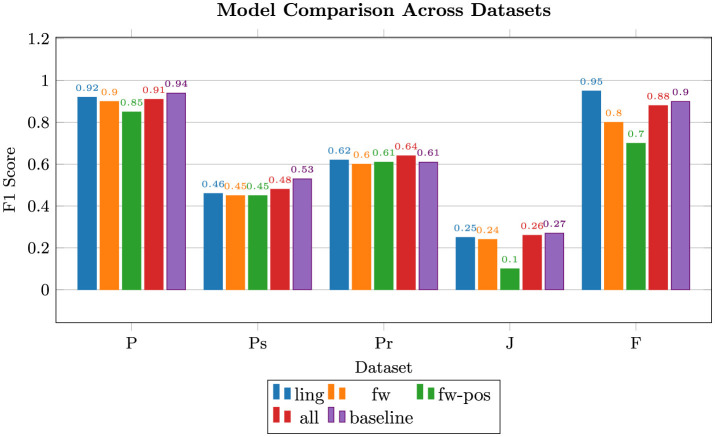
Random Forest F_1_ scores for the five feature types: linguistic (ling), function words (fw), pos tags of function words (fw-pos), combination of the three (all), and unigram tf-idf (baseline); F, fake news; J, job scams; P, phishing; Pr, product reviews; Ps, political statements.

**Figure 3:**
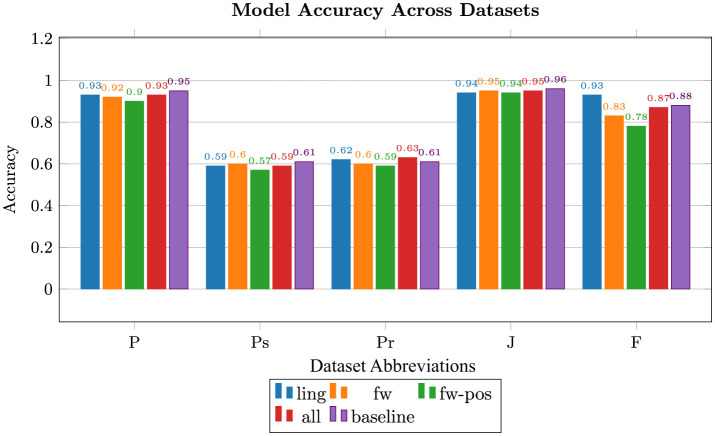
Random Forest accuracies for the five feature types: linguistic (ling), function words (fw), pos tags of function words (fw-pos), combination of the three (all), and unigram tf-idf (baseline); F, fake news; J, job scams; P, phishing; Pr, product reviews; Ps, political statements.

The accuracy and F_1_-scores of the model using each of the feature sets across the five datasets are shown in Figures 2, 3, respectively. It is important to note that Job Scams' data appear to be heavily imbalanced and the models' performance on it is not an ideal indicator of feature quality. Generally, the combined set of shared features is nearly on par with the baseline, with linguistic, function word, and function word POS following in the order given. Notable exceptions are Product Reviews where linguistic and combined features beat the others, including the baseline, and Fake News with linguistic features outperforming the rest by a significant margin. We hypothesize that the relative length and richness of news articles may be in part responsible for this phenomenon.

## 6 Deep-learning based experiments

To investigate the possible existence of other deception signals, we turn to deep learning. If universal deception signals exist, then a deep-learning model can learn to detect them. To determine whether this happens, we perform two experiments on the same five cleaned datasets of the previous section. First, we evaluate the performance of models trained on multiple domains. Then, we train models on one domain and evaluate their performance on other domains.

### 6.1 Model

Our model architecture consists of a base pre-trained transformer model, a dropout layer, and a linear layer. As standard in NLP, we prepend a [CLS] token to the text, pass the text through the base model, and perform classification on the last-layer embedding of the [CLS] token.

### 6.2 Multi-domain experiment

If deep-learning models trained on multiple domains pick up on universal deception signals, then we should expect performance on *individual* domains to be positively correlated among each other. Conversely, if they only learn domain-specific signals, then we should expect performance on individual domains to be negatively correlated with one another.

We train 100 models on the union of our datasets. We use a random 80/10/10 train/validate/test split for each dataset with uniformly drawn hyperparameters. We use BERT-base and RoBERTa-base for our base models, dropout percentages between 0.1 and 0.5, and the AdamW optimizer with learning rates between 0.00001 and 0.0001.

We then evaluate each model on the individual test sets. We exclude models that failed to converge and models that have an outlier F_1_ score using the IQR test and perform pairwise linear regression on the remaining F_1_ scores.

**Figure 4:**
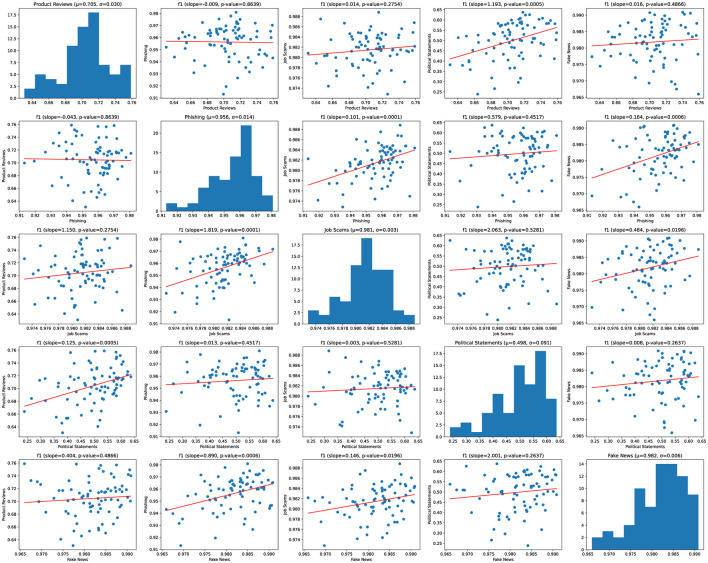
Pairwise F_1_ score scatter matrix of converged combined models. Outliers are excluded.

We present our results without outliers in Figure 4. All pairs of tasks except for product reviews and phishing are positively correlated, with five of them significant at the 0.05 level.

### 6.3 Cross-domain generalization experiment

If a deep-learning model primarily learns a universal deception signal, then it should generalize to deception domains that it has not yet seen. In particular, they should be able to achieve a higher F_1_ score than a coin flip classifier, which we can calculate using the formula CF F_1_ = *q*/(0.5+*q*), where *q* is the portion of the dataset that is deceptive.

On each dataset, we train 100 models with hyperparameters drawn from uniform distributions. We use BERT-base and RoBERTa-base for our base models and values between 0.1 and 0.5 for dropout percentage. For our learning rate, we use a different range for each task to minimize divergence: [0.00001, 0.00006] for product reviews, [0.00001, 0.000025] for job scams, [0.00001, 0.00010] for phishing, [0.00001, 0.00004] for political statements, and [0.00001, 0.00010] for fake news.

Each model is evaluated on each dataset, ignoring models that fail to converge. We perform a 1-sample *t*-test with the alternative hypothesis “the mean F_1_ in domain Y of models trained on X is less than or equal to the coin flip F_1_ of Y.” We report the resulting *p*-values in Table 3. In ten cases, models trained on one domain manage to beat the coin-flip baseline at a 0.01 significance level, with nine cases beating the coin-flip baseline at the 10σ (*p* < 7.62 × 10^−24^) level. However, we also find that eight pairs have a *p*-value of 1.00, meaning that they performed worse than the coin-flip baseline.^7^

**Table 3:** *p*-values in the cross dataset experiment.

**Dataset**	**Product**	**Phishing**	**Job**	**Political**	**Fake**
	**reviews**		**scams**	**statements**	**news**
Product reviews	0.00^†^	1.00	0.00^†^	1.00	1.00
Phishing	1.00	0.00^†^	0.00^†^	1.00	1.00
Job Scams	0.00^†^	0.98	0.00^†^	0.00^†^	0.00^†^
Political statements	1.00	1.00	0.00	0.00	0.96
Fake news	0.00^†^	0.00^†^	0.00^†^	0.00^†^	0.00^†^

Values below 0.01 are considered significant. A dagger indicates that the 0.00 values are correct to two decimal places. The 0.00 values without the dagger have a 0 in at least the third place after the decimal.

Interestingly, we also find that the fake news models manage to beat the coin flip on all domains. We suspect that this is due in part to its larger size but leave this as a direction for future research.

### 6.4 Discussion

The multi-domain experiment provides strong support for the existence of universal deception signals. All but one pair are positively correlated. Five are statistically significant, and the one negative correlation is not statistically significant. In contrast, the results of our cross-domain generalization experiment are mixed. While some pairs beat the coin-flip baseline, others performed worse than the baseline.

Taken together, these results suggest that both universal and domain-specific deception signals exist. Models trained on a single task will learn both universal and task-specific signals, potentially resulting in poor generalization to other deception domains. Therefore, training a domain-independent deception detector will likely require a diverse domain-independent dataset.

## 7 Cross-domain detection

We also built detectors for deception and conducted the following experiments (Zeng et al., 2022).

### 7.1 Single-task baselines

To evaluate the diversity and difficulty of our collected tasks, we fine-tuned BERT-base (Devlin et al., 2018) classification models on each of our datasets. As standard in NLP, our model adds a linear layer that generates the prediction from the final classification token embedding. We trained using the AdamW optimizer with a learning rate of 2 × 10^−5^, a batch size of 16, dropout of 0.1, and a random 80/10/10 train-val-test split. We trained for five epochs with early stopping on the validation set on NVIDIA V100 GPUs with automatic mixed precision. We then evaluated our models against all datasets using a TPU from Google Colab.^8^ If a dataset was used to train the model, we used the held-out test set. Otherwise, we evaluated against the full dataset.

We report the accuracies and F_1_-scores measured in Tables 4, 5. BERT performed well on the phishing, job scams, and fake news tasks, with F_1_ scores greater than 0.98. However, it performed poorly on product reviews and political statements, with F_1_ scores of 0.594 and 0.708, respectively. We suspect that this is due in part to the lengths of the texts; many product reviews and almost all political statements are only one or two sentences long. Interestingly, we find that product reviews and fake news transfer well to the Job Scams task, achieving F_1_ scores greater than 0.950, but not vice versa.

**Table 4:** Accuracies obtained on the cleaned datasets.

**CD**	**Cleaned datasets**				

	**Prod**	**Phish**	**Job**	**Pols**	**News**
Prod	**0.708**	0.723	0.915	0.555	0.625
Phish	0.521	**0.988**	0.436	0.537	0.560
Job	0.493	0.396	**0.965**	0.444	0.442
Pols	0.509	0.505	0.187	**0.640**	0.609
News	0.503	0.410	0.926	0.536	**0.997**

Emboldened numbers indicate accuracies measured on a held-out test set.

**Table 5:** F_1_ scores obtained on the cleaned datasets.

**CD**	**Cleaned datasets**				

	**Prod**	**Phish**	**Job**	**Pols**	**News**
Prod	**0.708**	0.555	0.955	0.095	0.301
Phish	0.444	**0.985**	0.594	0.124	0.021
Job	0.647	0.565	**0.982**	0.608	0.613
Pols	0.437	0.601	0.274	**0.594**	0.511
News	0.667	0.575	0.962	0.527	**0.997**

Emboldened numbers indicate F_1_ scores measured on a held-out test set.

### 7.2 Models for the combined dataset

We fine-tuned four models on the union of all our datasets using the same method, with an 80/10/10 train-val-test split for each individual dataset, and hyperparameters as our individual models. For our base models, we used BERT-base and RoBERTa (Liu et al., 2019) (110 million parameters), and the larger BERT-large and RoBERTa-large (340 million parameters) pre-trained models.

Surprisingly, the small base models performed better than the large models, with RoBERTa performing slightly better. BERT-base and RoBERTa-base achieved 0.904 and 0.904 F_1_ scores (the slight gap disappears on rounding), respectively, while their large counterparts achieved F_1_ scores of 0.882 and 0.900. However, when we break down performance by task (Tables 6, 7), we find that BERT-base performed better, achieving the highest F_1_ score in 3/5 tasks and was still close in performance for the other two tasks (to the winners in the individual task experiment and to the winners on combined).

**Table 6:** Combined model accuracies on individual tasks.

**Classifier**	**Cleaned datasets**				

	**Prod**	**Phish**	**Job**	**Pols**	**News**
BERT	**0.706**	0.973	**0.966**	**0.637**	**0.991**
RoBERTa	0.510	0.394	0.950	0.484	0.450
BERT (L)	0.677	**0.974**	**0.966**	0.571	0.983
RoBERTa (L)	0.511	0.420	0.958	0.452	0.442

Emboldened numbers indicate the highest perforomance on each dataset.

**Table 7:** F_1_ scores of the combined models on individual tasks.

**Classifier**	**Cleaned datasets**				

	**Prod**	**Phish**	**Job**	**Pols**	**News**
BERT	**0.706**	**0.967**	0.982	0.582	**0.990**
RoBERTa	0.660	0.561	0.974	0.589	0.620
BERT (L)	0.648	0.967	**0.982**	0.257	0.981
RoBERTa (L)	0.677	0.591	0.978	**0.618**	0.613

Emboldened numbers indicate the highest performance on each dataset.

### 7.3 Discussion

Our results show that a single model can recognize multiple forms of detection. For example, a BERT model has high accuracy/F_1_ scores on 3 out of 5 tasks and is still close to individual models on the other two. Another interesting result is that BERT and BERT(L) trained on the combined dataset beats in accuracy and F_1_ the individual BERT trained and tested on job scams dataset. RoBERTa could do it only for F_1_ on the Politics dataset.

## 8 Conclusion and future work

We have provided new definitions for deception based on explanations and probability theory. We gave a new taxonomy of deception that clarifies the explicit and implicit elements of deception.

We have argued against hasty conclusions regarding linguistic cues for deception detection and especially their generalizability. The critiques contained in Fitzpatrick et al. (2015), Vogler and Pearl (2020), and Vrij (2008) may present a valid point, namely that some linguistic cues might not generalize across the broad class of attacks. However, over-generalizations should be made with caution as they discourage future domain-independent deception research. Moreover, we have presented evidence showing that there do exist common linguistic cues in deceptive attacks with widely varying goals and topical content.

Our linguistic analysis of four datasets and cross-dataset analysis of five different deception datasets shows that there are linguistic features, some at the surface level and some deeper, that can be used to build classifiers for more general deception datasets. With all the new developments in machine learning and NLP, we believe that research on linguistic deception detection is poised to take off and could result in significant advances.

We propose three concrete directions for future work: (a) investigation of the domain pairs that underperform in cross-domain detection, (b) comparing our BERT/RoBERTa results with the latest BERT variants, e.g., the nBERT model (Rasool et al., 2025), and (c) exploring multimodal datasets that integrate different modalities, e.g., text, images, audio and video, and different domains of deception.

## Data Availability

Publicly available datasets were analyzed in this study. This data can be found here: https://zenodo.org/records/8371762.

## Footnotes

^1^ https://www.surveymonkey.com/curiosity/axios-january-6-revisited

^2^ https://dblp.org

^3^Searched on 17 February 2025.

^4^Note that the topics can vary in a heterogeneous application, such as fake news detection, since some items could be on sport and some on politics or religion. Moreover, the goals may or may not be different. Hence, we avoid the term “domain” to refer to applications such as fake news.

^5^All these DBLP search results are as of 17 February 2025.

^6^At https://zenodo.org/record/6512468#.ZBVRUhTMLQM.

^7^This negative transfer plus domain shift can be mitigated by a few modifications that include: (a) masking domain-specific tokens, (b) adapter-based DANN training (Ganin et al., 2016), (c) calibrated thresholds, and (d) reducing source dataset size for transfer (Triplett et al., 2025).

^8^In our experiments, we noticed slight differences in outputted predictions evaluating on TPUs vs. on GPUs due to differences in floating-point representations, but the differences were not statistically significant.

## Author's note

This is a thoroughly revised version of a 2023 arXiv draft containing substantial new material. Cross-domain detection results in this paper were presented in a poster at *ACM CODASPY 2022*.
