# Supplementary material for: Domain-independent deception: a new taxonomy and linguistic analysis
Source: Front Big Data. 2025 Sep 30;8:1581734. doi: 10.3389/fdata.2025.1581734 (PMC12521749; doi:10.3389/fdata.2025.1581734)
Supplement: Supplementary file 1 [file Presentation_1.pdf]

Table A1: Statistics of the four available datasets covering different domains.

**Dataset**	**Size**	**Truthful / Deceptive**	**Category**
Amazon Reviews	20,976	10,481 / 10,495	Fake reviews
DecOp	1,250	625 / 625	Deceptive opinions
IWSPA-AP	5,026	4,429 / 597	Phishing emails
WELFake	62,101	34,615 / 27,486	Fake news
**Total**	89,353	50,150 / 39,203	Combined

A Significance Testing of Linguistic Cues from Deception Literature

For significance-testing of the linguistic cues from the deception literature [76, 65], we did a preliminary analysis of these four datasets. DecOp [9] containing deceptive opinions, the WELFake dataset [65] containing fake news, the IWSPA-AP dataset [66] containing phishing/legitimate emails, and the Amazon Reviews dataset [24] consisting of truthful and fake product reviews. All are publicly available, but one of them, DecOp, is a small laboratory dataset with its limitations.

The goals in these datasets are quite diverse. Phishing email attackers wish to install malware or steal identity/money. Deceptive opinion/review authors wish to sway opinions on services or products. Fake news authors wish to sway elections, divide people, or cause chaos. Fake product reviews are designed to sell more of a certain product or depress the sales of competitors.

- The DecOp Dataset: This dataset is from [9]. It contains truthful/deceptive opinions on several topics such as abortion and cannabis legalization. These opinions were collected using crowdsourcing in the US and Italy. The researchers also trained transformer models that achieved 0.62–0.90 accuracies with different settings.
- The WELFake Dataset: This dataset is from [65]. It draws upon multiple true/fake news datasets.
- The IWSPA-AP Dataset: This is the IWSPA Anti-Phishing competition dataset of emails [66].
- The Amazon Reviews Dataset: This comprises real and fake Amazon reviews from a Kaggle repository [24].

Dataset statistics are shown in Table A1. Of these, the WELFake and Amazon Reviews Dataset are also included in the main sections.

We analyzed each dataset for any artifacts of data collection and cleaned them to remove such artifacts. The cleaning procedures include two parts: text removal and text cleaning. We removed a total of 10,728 duplicate, non-English, or empty bodies, giving a total of 89,353 items. We then sanitize the texts using the methods discussed in [73]. We remove meta-data in emails and source leaks in news and replace HTML break tags with new lines. Additionally, the authors of [73] found that the provided labels in WELFake [65] are flipped, so we flip its labels as a final cleaning step. We removed a total of 10,728 duplicate, non-English, or empty bodies, giving a total of 89,353 items.

A.1 Features: Linguistic Cues

We extracted 27 total textual features from the literature shown in Table A2. Features 1–27, with 15, 16 and 23 skipped, are from [75], and features 28–30 are from [65]. We then measure their values on the deceptive and legitimate samples of each dataset and count the number of datasets with statistically significant differences (using appropriate statistical tests). A difference is significant if and only if its p-value, after Bonferroni-Holm correction, is smaller than the threshold 0.01. Since there is some debate on the multiple comparisons issue (e.g., see [25]), we report statistically significant features both with and without the correction in Table A3.

Table A2: List of linguistic cues. Features 1–27 are from [75], and features 28–30 are from [65]. Features with an asterisk (∗) are selected after testing on the four available datasets. Features in **bold** remain qualified after adjusting their p-values per the Bonferroni-Holm method.

1. **words*** (words)	W(D). NLTK's word tokenizer [38] was used to identify words.	16. generalizing terms [skipped]	*Missing computational description.* The definition is: refers to a person (or object) as a class of persons or objects that includes the person (or object).
2. **verbs*** (verbs)	Num-verbs(D). NLTK's word tokenizer was used to identify verbs.	17. **self reference*** (self_ref)	Num-first person singular pronouns(D) (i.e., Num-$\{I, me\}$/W(D).
3. noun phrase	Num-noun phrases(D). The noun chunk function in spaCy [34] was used to identify noun phrases.	18. group reference	Num-first person plural pronoun(D) (i.e., Num-$\{we, us\}$)/W(D).
4. **sentence*** (sens)	S(D). NLTK's sentence tokenizer was used.	19. emotiveness	(Num-adj.(D) + Num-adv.(D)) / (Num-nouns(D) + Num-verbs(D)).
5. average number of clauses	The average number of clauses per sentence. Stanza [52] was used for POS tagging. Numclauses = Num-verb predicates (word.upos = 'VERB') − Num-root (word.deprel = 'root') − Num-conjugations (word.deprel = 'conj').	20. lexical diversity	Num-distinct words / W(D).
6. **average sentence length*** (sen_len)	W(D) / S(D)	21. content word diversity	Num-unique content words(D) / Num-content words(D). Content words are words with lexical meanings, as opposed to function words. Methods to identify content/function words are discussed in the Function Word n-gram section in the Appendix.
7. average word length* (word_len)	Num-characters(D) / W(D). Characters include digits, punctuation, and spaces.	22. redundancy* (redun)	Num-function words(D) / S(D).
8. average length of noun phrase (NP)	Num-words in noun phrases(D) / Num-noun phrase(D). Noun phrases are identified in the same way as in Feature (3).	23. typographical error ratio [skipped]	*This feature is skipped because exploratory analysis showed that the typographical error ratio is zero for most texts in both categories. The popularity of the auto-correct feature on browsers and text editing software has probably diminished the effectiveness of this feature.*
9. **pausality*** (paus)	Num-punctuation marks(D) / S(D)	24. spatiotemporal information	Num-('space' + 'time') / W(D), where 'space' and 'time' refer to the Num-words with the tag 'space' and 'time' in the LIWC2015 dictionary. LIWC is a dictionary that associates words with various tags; we used a commercial program from Pennebaker Conglomerates [63].
10. **modifier*** (modi)	Num-adjectives and adverbs(D). A word is an adjective or adverb iff word.upos = 'ADJ' or word.upos = 'ADV'. Stanza was used for POS tagging.	25. perceptual information	Num-'percep' / W(D). 'percep' is defined similarly, as in Feature (24).
11. modal verb* (modal)	Num-modal verbs(D) / W(D). A word is a modal verb iff word.upos = 'AUX' and word.xpos = 'MD'. Stanza was used for POS tagging.	26. positive affect	Num-'posemo' / W(D). 'posemo' is defined similarly, as in Feature (24).
12. certainty* (cert)	Num-words that have the tag 'certain' in the LIWC2015 dictionary(D) / W(D).	27. negative affect	Num-'negemo' / W(D). 'negemo' is defined similarly in Feature (24).
13. other reference	Num-third person pronoun(D) / W(D). A word is a third person pronoun iff word.xpos = 'PRP' and word.feats = 'Person=3'. We used Stanza for POS tagging.	28. Gunning fog grade readability index [5]	An index to quantify the readability of a text by estimating the years of education required to understand the text. We used TextSTAT [2] to calculate it.
14. passive voice	Num-passive voice verb(D) / W(D). A word is a passive voice verb iff word.deprel = 'aux:pass'. We used Stanza for POS tagging.	29. **SMOG readability index*** (smog)	Another index trying to estimate the years of education required to understand the text. We used TextSTAT to calculate this.
15. objectification [skipped]	*Missing computational description* [75]. It is defined as an expression given (as an abstract notion, feeling, or ideal) in a form that can be experienced by others and externalizes one's attitude.	30. automatic readability index* (ari)	Similar to Features (28) and (29). We also used TextSTAT for this.

Table A3: Behavior of features that show statistically significant differences between the truthful and deceptive classes. # *sig* is the number of datasets where the feature shows a significant difference. A positive number means the feature value is higher in the deceptive class. Emboldened features are those still qualified after the Bonferroni-Holm *p*-value adjustment.

	Feature	# sig		Feature	# sig
1	**word**	−3	8	modal verb	+3
2	**verb**	−3	9	certainty	+3
3	**sentence**	−3	10	**self ref**	−3
4	**sen_len**	−3	11	redundancy	−3
5	word_len	−3	12	**SMOG**	+4
6	**pausality**	−4	13	ARI	−3
7	**modifier**	−3			

A.2 Results: Selected Features

We find that the following features are statistically lower for deceptive samples in three or more datasets:[9] number of words, number of verbs, number of sentences, sentence length, word length, pausality (number of punctuation marks per sentence), number of modifiers (adjectives and adverbs), self reference (number of first-person plural pronouns), redundancy (number of function words per sentence), and the Automatic Readability Index.

We find that the following features are statistically higher for deceptive samples in three or more datasets: modal verbs, certainty (number of words that have the certainty tag in the LIWC 2015 Dictionary per word), and the SMOG readability index. We used Stanza [52] for POS tagging. Removing positive affect, since it is covered by our LIWC list of features below, gives us 13 features.

B Linguistic Patterns in Deceptive Language

The analysis reveals that deceptive texts differ systematically from truthful ones in multiple linguistic features. We offer theoretical explanations grounded in cognitive psychology, deception research, and linguistic analysis.

B.1 Cognitive Load Theory

Deceptive communication is cognitively demanding. Liars must construct a false narrative, monitor its consistency, and suppress the truth. To reduce this load, they often use simpler language.

- **Fewer words, verbs, and sentences**: Reflect lower narrative complexity.
- **Shorter sentences and words**: Reduce cognitive effort.
- **Fewer modifiers (adjectives/adverbs)**: Avoid over-specification.
- **Lower redundancy (function words)**: Conserve effort in crafting the lie.

B.2 Psychological Distance and Impression Management

Deceivers tend to distance themselves psychologically from the lie and avoid appearing personally invested.

- **Lower self-reference (first-person plural pronouns)**: Reduces association with the content.
- **Lower pausality (punctuation per sentence)**: Results in flatter, less expressive tone.

[9] The precise number of datasets with fewer is in Table A3.

B.3 Confidence Signaling

To appear credible, liars often overcompensate by projecting confidence and assertiveness.

- **Higher modal verbs and certainty words (LIWC)**: Create an illusion of decisiveness.
- **Higher SMOG Index**: Use of more complex or polysyllabic words to seem intelligent.

B.4 Readability Trade-off

Deceptive texts may exhibit lower **ARI** (suggesting simpler sentence structures) while showing higher **SMOG** due to sporadic complex word use for credibility.

B.5 Summary Table

We summarize the results of this analysis in Table A4.

Table A4: Linguistic differences observed in deceptive texts.

**Feature**	**Observation in Deception**	**Explanation**
Number of words, verbs, sentences	Lower	Simplifies cognitive effort
Sentence length	Lower	Easier to construct under pressure
Word length	Lower	Simpler vocabulary under cognitive load
Pausality (punctuation)	Lower	Less narrative expression
Modifiers (adj/adv)	Lower	Avoids over-specification
Self-reference	Lower	Creates distance from falsehood
Redundancy (function words)	Lower	Less structural complexity
Automatic Readability Index (ARI)	Lower	Simpler sentence structure
Modal verbs	Higher	Assertive tone projection
Certainty words (LIWC)	Higher	Impression of confidence
SMOG Index	Higher	Strategic complexity to sound credible

C Function-Word n-Grams

In this section, we describe our initial analysis of function word n-grams for the same four public datasets as in the previous section of the appendix.

C.1 Method

We combine function words and n-grams by looking for n-grams of function words that appear significantly more or less often in deceptive texts than truthful texts.

We first extract the function words from our texts using a list from the publicly-available PhishBench 2.0 [74] and compute function-word n-grams for n from one through eight (an example of this process is in Figure A1).[10]

We then calculate the frequency of occurrence of every n-gram x in each text t using the formula

$$Occ_n(x,t) = \sum_{s \in t} \frac{\#_x(s)}{|s| - n}$$

where $|s|$ denotes the number of words in sentence s of t, and $\#_x(s)$ denotes the number of times x occurs in s.

[10] We also explored POS tagging, which produced similar results at greater computational cost.

Table A5: Table of n-grams that show a consistent significant difference in Occ in $\# \ sig$ datasets. A positive $\# \ sig$ means the n-grams is generally more frequent in deceptive texts. Emboldened unigrams are still qualified after the Bonferroni-Holm correction.

	n-gram	# sig		n-gram	# sig
1	**I**	+3	11	their	+3
2	**they**	+3	12	**if**	+3
3	out	+3	13	both	+3
4	is a	−3	14	at the	+3
5	**only**	+3	15	**will**	−3
6	do	+4	16	**me**	+3
7	**at**	+3	17	**but**	+4
8	about	+3	18	than	+3
9	**that**	+3	19	**and**	−3
10	**them**	+3	20	for	−3

We then ranked the function word n-grams by the difference of the aggregate occurrences in the two classes (truthful versus deceptive) and selected the top 100 for statistical significance testing. We then ran two-sample t-tests on each dataset, comparing occurrence scores between legitimate and deceptive texts, and identified ones that showed a consistent significant difference, both with and without Bonferroni-Holm correction.

C.2 Results

The results are in Table A5. Only two n-grams, "do" and "but" show significant differences between truthful/deceptive groups in all four datasets. Both unigrams ($n = 1$) are more frequent in deceptive texts. Twenty n-grams, including "I", "they," "is a," and "at the," show significant differences between truthful and deceptive texts in three out of four datasets. After Bonferroni-Holm correction, 11 n-grams still qualified.

An individual dataset has thousands of FW n-grams, of which between 100 to 400 of them are significant discriminators of deceptive texts. In our experiments, 20 terms, 18 of them unigrams, show a common behavior across at least three out of the four datasets, and four across all four. After Bonferroni-Holm correction, 11 out of 20 are still qualified. We also notice that the lower the n, the higher the chance that the n-gram will show a significant difference between truthful and deceptive groups.

D Linguistic Signals of Deception: A Focus on n-grams

In this section, we provide theoretical explanations for findings related to n-gram frequency patterns in deceptive versus truthful texts. The focus is on the observation that certain unigrams, particularly "do" and "but," consistently appear more frequently in deceptive samples across multiple datasets.

D.1 Emphatic and Deflective Functions of "do" and "but"

"do" often serves to reinforce a statement:

- E.g., "I **do** know her." — an emphatic assertion of truthfulness.

"but" functions as a contrastive conjunction:

- E.g., "I was late, **but** it wasn't my fault." — common in excuse-making and blame deflection.

These uses align with the behavioral strategies of deceivers who aim to sound credible and mitigate suspicion.

D.2 Psychological Distance and Function Words

Function words (e.g., pronouns, articles, prepositions) are processed automatically and less consciously controlled, making them excellent indicators of underlying psychological states.

- Deceptive texts often show **lower self-reference** ("I") and **higher distancing** ("they").
- These patterns are consistent across domains and datasets, reinforcing their generalizability.

D.3 Summary Table

We summarize the theoretical explanations in Table A6.

Table A6: Explanations for consistent n-gram patterns in deceptive texts.

**Finding**	**Linguistic Pattern**	**Theoretical Explanation**
High frequency of "do"	Emphatic assertion	Reinforces truth-claims
High frequency of "but"	Contrast/justification	Deflects responsibility
FW n-grams are significant	Psychological leakage	Less conscious control
Cross-dataset robustness	Shared linguistic signals	Domain-independent deception markers

E LIWC and BERTAA Features

The 55 stylistic features from [18] are listed below: .

- Length of text: len_text
- Number of words: len-words
- Average length of words: avg-len
- Number of short words: num-short-w
- Proportion of digits and capital letters: per_digit, per_cap
- Individual letters and digits frequencies: f_a, f_b, f_c, f_d, f_e, f_f, f_g, f_h, f_i, f_j, f_k, f_l, f_m, f_n, f_o, f_p, f_q, f_r,f_s, f_t, f_u, f_v, f_w, f_x, f_y, f_z, f_0, f_1, f_2, f_3, f_4, f_5, f_6, f_7, f_8, f_9
- Hapax-legomena: richness
- Frequency of 12 punctuation marks: f_e_0, f_e_1, f_e_2, f_e_3, f_e_4, f_e_5, f_e_6, f_e_7, f_e_8, f_e_9, f_e_10, f_e_11

LIWC denotes the Linguistic Inquiry and Word Count program. It uses dictionaries of words that fit into different categories like tone_pos (positive tone) to identify words in the text that fall into that category (happy, elated, excited, . . .) and reports *percentage of words in the text* that fall into those categories except for WC (word count) and WPS (words per sentence). More information about LIWC can be found at `https://www.liwc.app/help`.

The 86 LIWC features that we used along with their abbreviations are as follows:

- Summary variables (8 features): Word count – WC, Analytical thinking – analytic, Clout – clout, Authentic – authentic, Emotional tone – tone, Words per sentence – WPS, Big words – BigWords, Dictionary words – Dic

- Linguistic simension (17 features): Total function words – function, Total pronouns – pronoun, Personal pronouns – ppron, 1st person singular – I, 1st person plural – we, 3rd person plural – they, Impersonal pronouns – ipron, Determiners – det, Articles – article, Numbers – number, Prepositions – prep, Auxiliary verbs – auxver, Adverbs – adverb, Conjunctions – conj, Negations – negate, Common verbs – verb, Quantities – quantity
- Psychological processes (28 features): Drives – drives, Affiliation – affiliation, Power – power, Cognition – cognition, All-or-none – allnone, Cognitive processes – cogproc, Insight – insight, Causation – cause, Discrepancy – discrep, Tentative – tentat, Certitude – certitude, Differentiation – differ, Affect – affect, Positive tone – tone_pos, Negative tone – tone_neg, Emotion – emotion, Positive emotion – emo_pos, Negative emotion – emo_neg, Anxiety – emo_anx, Sadness – emo_sad, Social processes – Social, Social behavior – socbehav, Prosocial behavior – prosocial, Politeness – polite, Moralization – moral, Communication – comm, Social referents – socrefs, Friends – friend
- Punctuation (5 features): All Punctuation – allpunc, Apostrophes – apostro, Periods – period, Commas – comma, Other punctuation – OtherP
- Expanded dictionary (28 features): Culture – culture, Politics – politic, Ethnicity – ethnicity, Technology – tech, Lifestyle – lifestyle, Home – home, Work – work, Religion – relig, Physical – physical, Health – health, Mental health – mental, Need – need, Lack – lack, Fulfilled – fulfill, Risk – risk, Curiosity – curiosity, Allure – allure, Perception – perception, Attention – attention, Motion – motion, Space – space, Visual – visual, Auditory – auditory, Feeling – feeling, Time – time, Past focus – focuspast, Present focus – focuspresent, Future focus – focusfuture

Note that a few of the features between these three lists: BERTAA, the 13 selected features from Table A3 and LIWC list are duplicates. Specifically, WD from LIWC, word in Table A3, and len_text from BERTAA are duplicative, and WPS from LIWC, average sentence length in Table A3 form another duplicate group. These are removed by the colinearity check mentioned in the feature analysis section, Section 5.

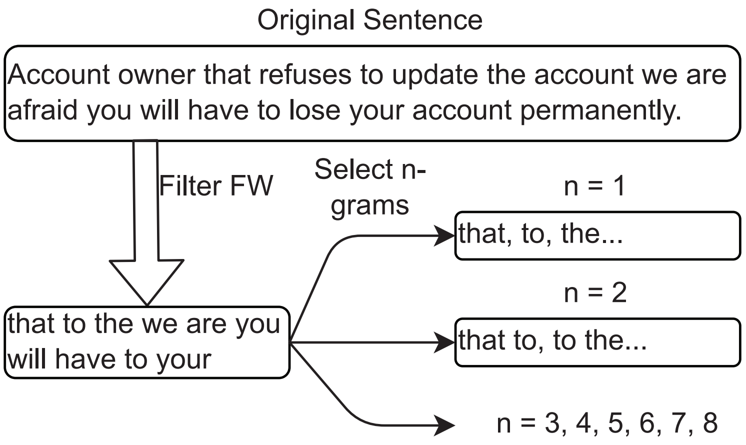

Figure A1: An example of extracting function-word (FW) n-grams from a sentence.
